# Utilization of whole genome sequencing for resolution of discrepant *Mycobacterium tuberculosis* drug susceptibility results: A case report^☆^

**DOI:** 10.1016/j.idcr.2021.e01308

**Published:** 2021-10-08

**Authors:** Susan Realegeno, Oladunni Adeyiga, Drew J. Winston, Omer E. Beaird, Omai B. Garner, Shangxin Yang

**Affiliations:** a UCLA Clinical Microbiology Laboratory, Department of Pathology & Laboratory Medicine, University of California, Los Angeles, Los Angeles, CA, USA; b Division of Infectious Diseases, Department of Medicine, University of California, Los Angeles, Los Angeles, CA, USA; c Division of Hematology and Oncology, Department of Medicine, University of California, Los Angeles, Los Angeles, CA, USA

**Keywords:** *Mycobacterium tuberculosis*, Whole genome sequencing, Drug resistance prediction, DST

## Abstract

A 44-year-old woman undergoing therapy for acute promyelocytic leukemia (APL) developed disseminated tuberculosis. *Mycobacterium tuberculosis* (TB) was isolated from the blood and sputum. Initial drug susceptibility testing (DST) of the blood isolate revealed resistance to isoniazid and ethambutol but the sputum isolate showed no resistance. Due to drug resistance concerns, the patient was treated with multiple second and third-line drugs, and suffered from drug side effects. To further investigate the DST discrepancies, whole genome sequencing (WGS) was performed on both isolates. No known resistance mutations to first line or second line drugs were identified in either isolate, which was confirmed by additional susceptibility testing performed by a different reference laboratory and the California Department of Public Health (CDPH) laboratory. Treatment was reduced to a simpler and less toxic regimen due to these investigations. WGS is shown to be a valuable tool for resolving discordant phenotypic DST results of TB isolates and has the potential to provide accurate and timely results guiding appropriate therapy in the clinical setting.

## Introduction

Reactivation of latent tuberculosis (TB) accounts for most of the TB cases in the United States [1]. The proportion of TB patients with complexities has increased over the years and treatment can be complicated by comorbidities and immunosuppressive therapies. Therefore, timely diagnosis and drug susceptibility results are essential for initiating optimal treatment. Isolation of mycobacteria requires specialized media and lengthy incubation (approximately 2 weeks or longer). Several methods for phenotypic drug susceptibility testing (DST) are available for first line drugs (rifampin, isoniazid, pyrazinamide, and ethambutol), however, all methods require additional incubation time (>1 week). In some cases, susceptibility testing for secondary drugs is also needed, further extending the time to receiving results. In recent years, whole-genome sequencing (WGS) has been shown to reliably predict TB drug resistance and can be done in a timely and cost effective manner [2].

Here, we present a challenging case of disseminated TB in a cancer patient. The initial erroneous phenotypic DST results led to unnecessary treatment with toxic and less effective non-first line drugs. WGS was utilized to resolve the discrepancies and ultimately helped guide the optimal treatment.

## Case

**Fig. 1:**
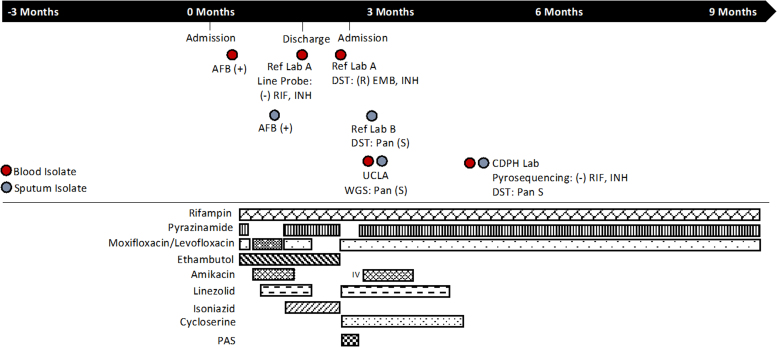
Clinical history and microbiological findings.

A 44-year-old female with a history of incompletely treated latent TB was diagnosed with acute promyelocytic leukemia (APL). She was treated with arsenic trioxide plus all-trans retinoic acid. She also received dexamethasone for suspected pulmonary differentiation syndrome. Her initial treatment was complicated by neutropenic fever, hepatoxicity, and suspected acalculous cholecystitis. She later underwent a cholecystectomy tube replacement with subsequent tube removal. Approximately one month after her APL diagnosis, she was transferred to our facility for a higher level of care. Blood cultures obtained as part of evaluation for neutropenic fever were positive for *Mycobacterium tuberculosis* (TB); follow up respiratory cultures were also positive for TB. A bone marrow biopsy showed multiple granulomas. Computed tomography (CT) imaging of the chest/abdomen/pelvis demonstrated diffuse lymphadenopathy and a pericardial effusion. Due to abnormal liver function tests, a liver biopsy was performed and showed multiple foci of confluent coagulative necrosis. In addition to receiving empiric antimicrobials for neutropenic fever, initial TB treatment was adjusted for her liver disease and included rifampin (RIF), pyrazinamide (PZA), moxifloxacin (MFX), and ethambutol (EMB) (Fig. 1). Given concern for liver toxicity, PZA and MFX were changed to levofloxacin, amikacin and linezolid (LZD). When her liver enzymes improved, isoniazid (INH) and PZA were added, and levofloxacin was changed back to MFX. When her neutropenia resolved, she was discharged home on RIF, INH, PZA, EMB, LZD, and MFX [3] despite persistent fevers which were attributed to widely disseminated TB with potential TB immune reconstitution inflammatory syndrome (IRIS).

The blood isolate was sent to Reference Lab A for DST by agar proportion method and rapid molecular multi-drug resistant TB screen by line-probe assay. While the phenotypic DST results were pending, the line-probe assay showed no mutations in *rpoB*, *katG*, or *inhA* targeted regions, suggesting susceptibility to RIF and INH (Table 1). Therefore, moxifloxacin and linezolid were stopped. However, 10 days later, the phenotypic DST results returned and showed resistance to EMB and INH, but susceptibility to RIF and PZA. In the meantime, the patient was re-admitted to the hospital for persistent fevers and congestive heart failure, which improved with diuresis. Continued fevers were attributed to disseminated TB and TB IRIS. The persistent fevers, the need to resume APL treatment, and the drug resistance profile of the blood isolate prompted the treatment team to switch to a 5-drug regime: para-aminosalicylic acid (PAS), cycloserine, LZD, RIF and levofloxacin. The patient could not tolerate PAS which was then replaced by PZA and amikacin, leaving the regimen to include 6 drugs: PZA, RIF, cycloserine, LZD, levofloxacin, and amikacin [3].

**Table 1:** TB drug susceptibility results.

Antibiotic	Performing Lab^a^	Technique	Target Regions	Blood	Sputum
Rifampin	Reference Lab	Culture-based		S	S
		Line Probe	*rpoB*	S	–
	UCLA	WGS	*rpoB, rpoC*	S	S
	Public Health Lab	Culture-based		S	S
		Pyrosequencing	*rpoB* (426–440), (441–452), 170	S	S
Isoniazid	Reference Lab	Culture-based		R	S
		Line Probe	*katG, inhA*	S	–
	UCLA	WGS	*katG, katG promoter, inhA, inhA promoter, aphC, aphC promoter, kasA, fabG1*	S	S
	Public Health Lab	Culture-based		S	S
		Pyrosequencing	*katG, inhA, ahpC, fabG1*	S	S
Ethambutol	Reference Lab	Culture-based		R	S
	UCLA	WGS	*embB, embA, embA promoter, embC, embR*	S	S
	Public Health Lab	Culture-based		S	S
Ethionamide	Reference Lab	Culture-based		R	–
	UCLA	WGS	*ethA, ethR, inhA promoter, inhA*	S	S
	Public Health Lab	Culture-based		S	S
Pyrazinamide	Reference Lab	Culture-based		S	S
	UCLA	WGS	*pncA, pncA promoter, rpsA, panD*	S	S
	Public Health Lab	Culture-based		S	S
Streptomycin	Reference Lab	Culture-based		S	S
	UCLA	WGS	*rrs, rpsL, gidB*	S	S
PAS	Reference Lab	Culture-based		S	–
	UCLA	WGS	*thyA, folC, ribD, folC, thyX*	S	S
Amikacin	Reference Lab	Culture-based		S	–
	UCLA	WGS	*rrs*	S	S
	Public Health Lab	Culture-based		S	S
Capreomycin	Reference Lab	Culture-based		S	–
	UCLA	WGS	*rrs, tlyA, idsA2*	S	S
	Public Health Lab	Culture-based		S	S
Kanamycin	Reference Lab	Culture-based		S	–
	UCLA	WGS	*rrs, eis promoter*	S	S
Cycloserine	Reference Lab	Culture-based		S	–
	UCLA	WGS	*ald, alr*	S	S
Clofazimine	Reference Lab	Culture-based		S	–
	UCLA	WGS	*Rv0678*	S	S
Linezolid	Reference Lab	Culture-based		S	–
	UCLA	WGS	*rplC, rrl*	S	S
Levofloxacin	Reference Lab	Culture-based		S	–
	UCLA	WGS	*gyrA, gyrB*	S	S
Moxifloxacin	Reference Lab	Culture-based		S	–
	UCLA	WGS	*gyrA, gyrB*	S	S
	Public Health Lab	Culture-based		S	S
Delamanid	UCLA	WGS	*fbiA, ddn, fgd1*	S	S
Ofloxacin	UCLA	WGS	*gyrA, gyrB*	S	S
Bedaquiline	UCLA	WGS	*Rv0678*	S	S

a Blood DST performed at Reference Lab A (agar proportion method); Sputum DST performed at Reference Lab B (MGIT based method); (–): Not Performed.

The respiratory isolate (1 month post admission) was sent to Reference Lab B and was susceptible to all first-line drugs as determined by liquid broth (MGIT) method. To investigate the discrepancy observed in DST results between the blood and sputum isolates, WGS was performed (Table 1). WGS revealed both isolates were of the Euro-American (LAM) lineage 4 and closely related to pan-drug sensitive TB CTRI-2 strain [4]. Drug resistance prediction analysis using TB-profiler [5], [6] and ResFinder [7] showed no known mutations to confer resistance to all first and second-line drugs (Table 1). Single nucleotide variant (SNV) analysis using CLC Genomics Workbench v12.0 (Qiagen, Germany) was performed using CTRI-2 (NCBI Reference Sequence: NC_017524.1) as the reference genome and showed only 5 SNVs between the blood and sputum isolate, confirming these two isolates are of the same lineage. Generally, the rate of change is 0.5 SNPs per genome per year and highly related isolates have within 5 SNPs differences [8]. None of the 5 SNVs were within any drug resistance related genetic regions. The results of the WGS investigation were communicated to the California Department of Public Health (CDPH) TB Control team, who requested both isolates sent to the CDPH laboratory for confirmatory phenotypic DST and pyrosequencing. The results confirmed both isolates were susceptible to all first and second-line drugs, and no mutations were identified in the targeted regions for RIF and INH. At this point, 4 months had passed from the initial positive blood culture of TB, and treatment regime was reduced to 3 drugs: RIF, PZA, and levofloxacin for another 5 months. Patient successfully completed the treatment and achieved resolution of fever without the use of anti-inflammatory medications.

## Discussion

Phenotypic DST for TB is currently considered the standard for determining drug resistance, but it is a lengthy procedure requiring a single isolate to be incubated in the presence of drug for several weeks to evaluate bacterial growth compared to growth without drug [9], [10]. Agar proportion or liquid culture are the two commonly used DST methods. Both require bacteria growing at a critical concentration, which is defined as the lowest concentration that inhibits 99% of "wild type" strains of TB *in vitro* that have not been exposed to the drug [11]. However, the critical concentration is hard to standardize and varies significantly depending on the method or media type used, leading to a lack of standardization among reference laboratories [12]. In addition, DST is prone to random lab errors that can lead to discrepant results [13]. In our case, the erroneous results from the first reference laboratory could be due to cross-contamination or isolate mix-up. Traditionally, questionable DST results require repeat testing, but the inherent lengthy turn-around-time usually causes further delay, and in this case, patient harm.

As demonstrated in this case report, the false resistance DST results prevented selection of optimal drug treatment and led to the unnecessary use of toxic second or third-line drugs. Previous studies have shown that WGS is able to reliably predict susceptibility to first and second-line drugs with high accuracy and negative predictive values above 98.5% [2], [14], [15]. WGS is the most comprehensive molecular method for detection of drug resistance determinant mutations with a relatively short turnaround time and can be a financially feasible option in laboratories performing sequencing [16], [17]. Cost for performing WGS is estimated to be 7% lower compared to routine microbiologic testing methods and the overall cost can be further decreased with higher sample throughput [17]. Although cost can vary depending on instrument, volumes, and reagents, more cost-effective options for lower-throughout laboratories have become available as sequencing technologies improve [18], [19]. Additional cost savings for performing WGS can be considered in the reduced hands-on time, streamlining of laboratory testing, and increased turn-around-time of actionable results compared to routine testing methods. In one laboratory, WGS results were available on average 9 days earlier for first-line drugs and 32 days earlier for second-line drugs compared to phenotypic results [16], allowing optimized patient care to be initiated sooner.

Ultimately, we were able to use WGS to provide strong evidence of pan-drug susceptibility in the TB isolates from the patient and resolved the discrepancies. If performed as early as possible, WGS would have provided the correct susceptibility results early in the treatment course and thus avoided the erroneous DST results and unnecessary patient harm due to the toxicity of sub-optimal drugs. Our report demonstrated a proof of concept for the usefulness of performing WGS for TB drug susceptibility prediction in the clinical setting.

## Ethical approval

Not applicable

## Consent

Patient written consent has been obtained.

## CRediT authorship contribution statement

**Shangxin Yang, Susan Realegeno:** Study design. **Susan Realegeno, Oladunni Adeyiga, Drew J. Winston, Omer E. Beaird:** Data collections. **Susan Realegeno, Shangxin Yang:** Data analysis. **Susan Realegeno, Shangxin Yang:** Writing. **Oladunni Adeyiga, Drew J. Winston, Omer E. Beaird, Omai B. Garner:** Review and edition.

## Conflict of interest statement

All authors declared no conflict of interest.
